# A Rare Case of Renal Cell Carcinoma Metastasis to the Coracoid Process

**DOI:** 10.7759/cureus.97677

**Published:** 2025-11-24

**Authors:** Minesh Patel, Aniket Deshpande

**Affiliations:** 1 Urology, Colchester General Hospital, East Suffolk and North Essex NHS Foundation Trust, Colchester, GBR

**Keywords:** coracoid process, health surveillance, renal cell metastasis, urologic oncology, urology and oncology

## Abstract

A 51-year-old man attended the emergency department with lower left abdominal pain and fever for three days. He recovered and was discharged home with oral antibiotics and a CT colonoscopy organised as an outpatient. This had incidentally demonstrated a solid mass in the patient’s left kidney in keeping with a renal cell carcinoma (RCC). The tumour was staged as pT2N0M0 and the patient underwent an uncomplicated open left radical nephrectomy. The RCC was classified as clear cell from histology. The patient was then kept on the biannual renal surveillance protocol. Six years after the operation, the patient had reported a five-month history of pain and restricted movement in his right shoulder. MRI findings were suggestive of a metastatic lesion within the coracoid process. An ultrasound-guided biopsy concluded the lesion to be a metastatic clear cell carcinoma, which originated from the patient’s RCC. A marginal resection of the lesion was performed at a tertiary centre with a good outcome. This represents a rare, reported incident of RCC metastasis to the coracoid process, an exceedingly uncommon site of spread.

## Introduction

Renal cell carcinoma (RCC) is the most common renal malignancy that accounts for 3% of all cancers in the adult population [1]. RCC is predominant in men with a peak incidence in the 6th and 7th decades of life [2]. However, an increasing incidence has been reported worldwide with a notable rise in younger patients [3]. The histology of RCC can be divided into clear cell and non-clear cell, the latter including papillary and chromophobe subtypes. The classic triad of visible haematuria, flank pain and a palpable mass in the abdomen is noted in only 6-10% of cases and therefore considered to be rare [4].

RCC is now largely diagnosed incidentally on imaging to investigate for other pathology within the abdomen. This has led to stage migration to the earlier stages over recent decades. On CT, RCCs often appear as a solid mass that arises from the renal cortex, distorting the contour of the kidney. A heterogeneous density is shown on imaging with multiple areas of necrosis and haemorrhage that enhance significantly following the administration of contrast unlike simple cysts. Margins may be irregular or well circumscribed. Calcification is visualised in certain subtypes. More advanced RCC can be shown to progress into the renal vein or inferior vena cava. RCCs differ from angiomyolipomas on imaging in that they distinctly lack macroscopic fat.

RCC has a strong propensity for haematogenous spread due to its rich vascularity. Common metastatic sites include the lungs, liver, brain and the bones. More specifically, the spine, pelvis and proximal femur are frequent sites of metastases throughout the skeleton [5]. Metastasis to the coracoid process is exceedingly rare, likely due to its relatively small size and lower vascular supply in comparison to the other aforementioned osseous structures. 

The timely recognition of atypical metastasis patterns is clinically relevant as such presentations affect diagnostic pathways, surgical planning and the overall diagnosis. We present the rare case of RCC metastasis to the coracoid process of the shoulder. 

This article was previously presented as a meeting abstract at the ASiT Innovation Summit in November 2021.

## Case presentation

We present a 51-year-old man who attended the emergency department seven years ago with lower left quadrant abdominal pain and fever for three days. The pain was sharp and localised in nature. He denied any nausea, vomiting, urinary symptoms or changes to his bowel movements. At the time of presentation, he had a relevant past medical history of reflux oesophagitis for which he was taking lansoprazole. On examination, the abdomen was soft with marked tenderness at the lower iliac fossa region. Blood tests showed a raised C-reactive protein (CRP) level of 119 (normal <5mg/L). The initial provisional diagnosis was diverticulitis. Subsequently, he was discharged with co-amoxiclav for seven days and a CT colonoscopy was scheduled within the next four weeks.

**Figure 1:**
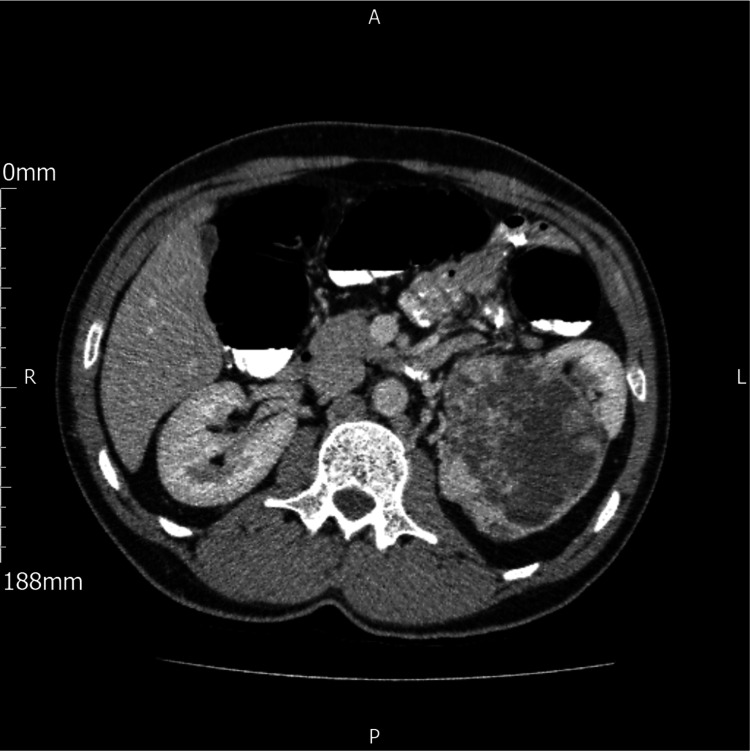
Cross-sectional image from CT colonography. A solid mass can be visualised in the left kidney.

**Figure 2:**
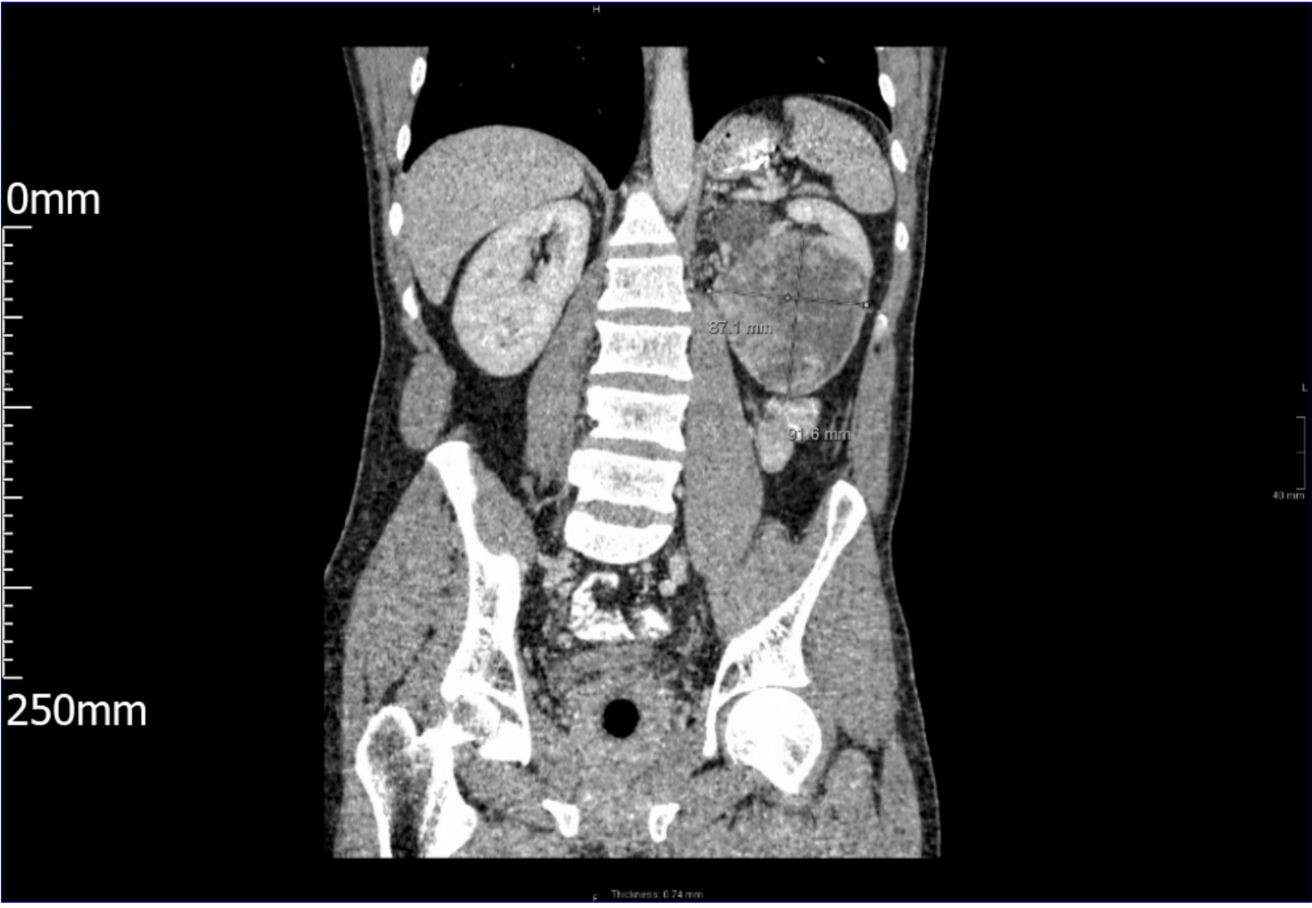
Longitudinal cross-sectional image from CT colonography.

CT colonoscopy showed no evidence of colorectal cancer or a clinically significant polyp. However, diverticular disease was noted in the distal descending and sigmoid colon. Most remarkably, a solid mass measuring 9.3cm by 8.2cm at its maximum craniocaudal and transverse diameter from the lower pole of the patient’s left kidney was reported (Figure 1 and Figure 2).

This was shown to obstruct the calyces within the upper pole of the kidney. There was no evidence of a tumour thrombus within the left ipsilateral renal vein and the inferior vena cava appeared patent. To add, there were no lytic bone lesions nor renal hilar or para-aortic lymphadenopathy. The differential diagnoses were RCC and an oncocytoma. 

This patient's tumour was staged as pT2N0M0 and the patient underwent an open left radical nephrectomy with no complications. Subsequently, he went on to make a full recovery. Histological analysis revealed that the mass was a clear cell RCC (G1pT2) with no sarcomatoid features. His Leibovich score was 3 which placed the patient in the intermediate risk of developing a metastasis from his RCC. He was then reviewed every six months according to the renal surveillance protocol.

Six years after the operation, our patient went to visit his GP regarding a five-month history of intermittent pain and restricted movement in his right shoulder. On examination, a palpable mass around his coracoid process was noted.

Investigations

**Figure 3:**
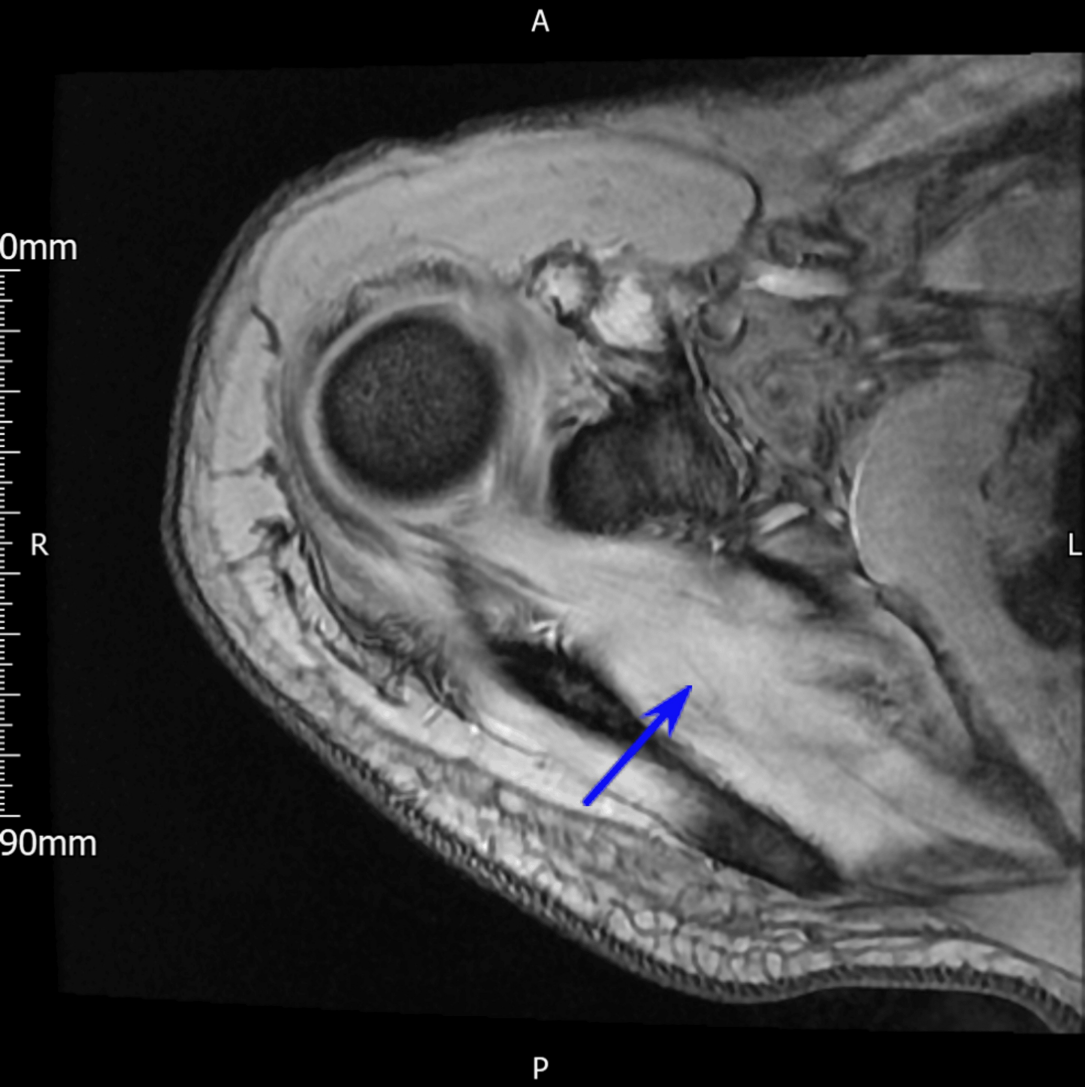
MRI axial 3D imaging of the coracoid mass. Arrow added to pinpoint the osseous mass.

He was then referred for an MRI scan of the shoulder (Figure 3). Interestingly, an abnormal signal was noted in the anterior portion of the coracoid with some loss of definition in the cortex inferiorly. In addition, there was an intermediate T1/high T2 signal mass extending inferiorly towards the subcoracoid recess measuring approximately 27mm by 30mm by 17mm. The radiologist’s suspicion was that the lesion was rather in line with an expansile osseous mass instead of a primary soft tissue mass lesion with secondary osseous invasion. The differentials included a metastatic lesion, myeloma or a primary osseous neoplasia. Furthermore, there was no evidence of a rotator cuff tendon tear or retraction and the muscular bulk appeared maintained around the shoulder girdle. A mild subacromial bursal effusion was noted. In light of this, the findings were relayed to the sarcoma orthopaedic team at a tertiary centre. To further characterise the lesion, an ultrasound (US)-guided biopsy of the site was arranged.

**Figure 4:**
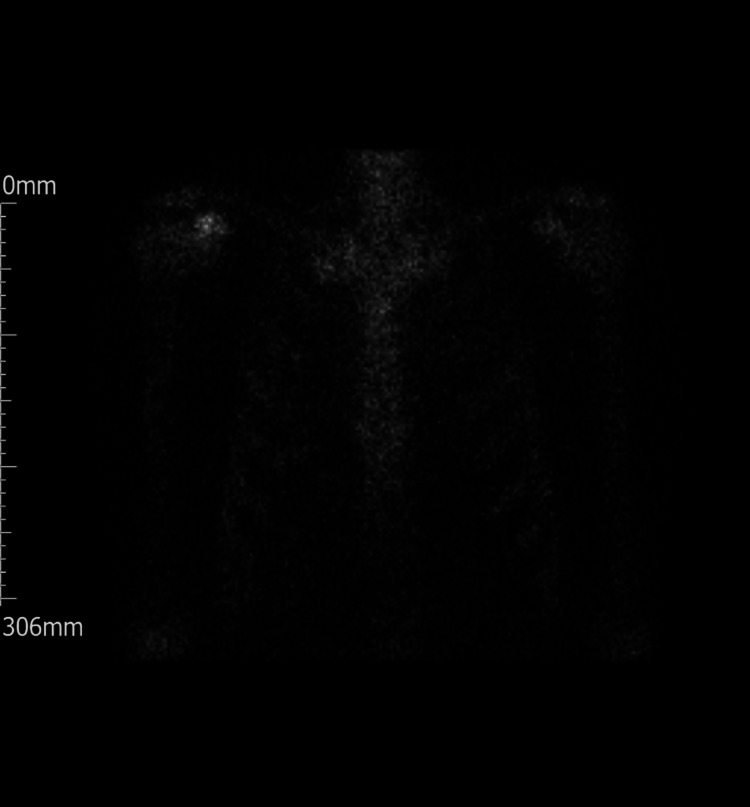
NM bone scan. Note the increased uptake in the right shoulder region. NM: Nuclear medicine

Unfortunately, histological analysis reported that the lesion was in fact metastatic clear cell carcinoma in line with the patient’s renal primary. The patient then underwent staging CT chest/abdomen/pelvis to investigate for any other metastases. This scan was also compared to his previous follow-up CT scans and reported as stable in appearance. Secondly, a nuclear medicine (NM) bone SPECT CT scan was performed (Figure 4). This showed that the lesion of the right coracoid was the single site of abnormality with no other osteoblastic metastases demonstrated from the whole body planar bone scan.

Treatment

After reviewing the imaging, surgical options were discussed in length with the patient. This comprised resecting the lesion with extensive reconstruction of the conjoint tendon, and curettage. Due to the absence of systemic disease progression as evidenced by the imaging, systemic therapy in the form of TKI targeted therapy or immunotherapy was not recommended. The patient agreed to surgery and a marginal resection of the lesion was performed.

Outcome & follow-up

A good macroscopic clearance of the lesion was noted from the surgery with no complications. The patient then went on to recover with the function of their shoulder grossly intact. A shoulder MRI has been scheduled by the orthopaedic surgeon in a few weeks as part of their follow-up. In addition, the oncologist has organised a CT chest/abdomen/pelvis for five months after the surgery.

## Discussion

RCC is the most common type of renal malignancy that derives from the epithelia of the proximal convoluted tubules. RCC accounts for 3% of all malignancies in the adult population and has an overall five-year survival rate of 45% [2]. However, in cases of metastatic RCC, the five-year survival rate plummets to 10% [6].

Overall, a third of patients with RCC present with metastatic disease at the time of diagnosis. Furthermore, in those patients with disease initially organ-confined, a significant proportion will go on to develop metastases. According to a study of 11,157 patients with metastatic RCC from the National Inpatient Sample in the United States, the most frequent sites of metastasis were lungs (45%), then bone (30%), lymph nodes (22%), liver (20%) and the adrenals (9%) [7]. More specifically, common skeletal sites implicated in RCC include the spine, pelvis and proximal femur. Involvement of the skeleton broadly presents with localised pain, restricted movement, fractures, hypercalcaemia of malignancy or most seriously, spinal cord compression [2]. As evident in our patient, the management of skeletal metastases warrants a multidisciplinary input from the urologist, an orthopaedic surgeon, oncologist, pathologist and the radiologist. Cases are largely managed using a combination of surgical resection, systemic therapy and targeted radiotherapy. In general, surgical resection is offered to remove the metastatic lesion, which may invade the surrounding tissues if left over time and compromise function.

The coracoid process is regarded as the “lighthouse of the shoulder” due to its close association with multiple tendons, ligaments and key neurovascular structures, including the brachial plexus and both the axillary vessels [8]. A tumour of the coracoid process is rare and tends to be primary in nature [9]. From a search of the literature, we could gather only a few cases of metastasis to the coracoid process: two cases from breast carcinomas [10], one from hepatocellular carcinoma [11], basal cell carcinoma [12] and the other from prostate adenocarcinoma [13]. As such, we present an extraordinary case of RCC metastasis to the coracoid process.

From taking a detailed history, performing a thorough examination and conducting the relevant scans, a provisional diagnosis can be made from the multitude of plausible differentials. Any previous history of RCC should always raise the suspicion of metastatic disease among the differential diagnoses. The earlier the diagnosis of a metastatic lesion of RCC origin, the better the prognosis, as this can be excised in the majority of cases. This highlights the importance of a diagnostic biopsy, most pertinently in sites where primary malignancy is uncommon. The analysis of the sample gathered elicits important details such as the tumour subtype, histological grade and any features such as sarcomatoid differentiation that collectively influence the treatment plan. Delays in diagnosis could lead to systemic metastasis, which therefore reduces the overall and disease-specific survival rates.

## Conclusions

This case highlights a rare instance of RCC metastasis to the coracoid process, occurring many years after a nephrectomy for organ-confined disease. The patient had presented with shoulder pain and a palpable mass, which was promptly investigated with imaging and a biopsy. Following the marginal surgical resection, his pain had resolved and shoulder function was preserved, demonstrating a favourable outcome. 

Further to this, this case underscores the significance of long-term follow-up in patients with a history of RCC, even years after curative treatment. It illustrates that metastatic spread can occur to extremely rare and unexpected sites and highlights the unpredictable nature of RCC. Early recognition of new, unexplained musculoskeletal symptoms in this cohort of patients is important as timely imaging, biopsy and multidisciplinary action allow for effective management and preservation of function. Lastly, this case emphasises the essence of coordinated care between surgical, oncological and radiological teams to achieve optimal outcomes in managing isolated oligometastases from RCC. 
